# Xanthogranulomatous Endometritis: A Challenging Imitator of Endometrial Carcinoma

**DOI:** 10.1155/2007/34763

**Published:** 2007-06-07

**Authors:** A. Işın Doğan-Ekici, Alp Usubütün, Türkan Küçükali, Ali Ayhan

**Affiliations:** ^1^Department of Pathology, School of Medicine, Yeditepe University, 34755 Istanbul, Turkey; ^2^Department of Pathology, School of Medicine, Hacettepe University, 06100 Ankara, Turkey; ^3^Department of Obstetrics and Gynecology, School of Medicine, Hacettepe University, 06100 Ankara, Turkey

## Abstract

Xanthogranulomatous inflammation is a distinguished histopathological entity affecting several organs, predominantly the kidney and gallbladder. So far, only a small number of cases of xanthogranulomatous inflammation occurring in female genital tract have been described, most frequently affecting the endometrium and histologically characterized by replacement of endometrium by xanthogranulomatous inflammation composed of abundant foamy histiocytes, siderophages, giant cells, fibrosis, calcification and accompanying polymorphonuclear leucocytes, plasma cells and lymphocytes of polyclonal origin. We present a case of a 69-year-old female complained of post menopausal bleeding and weight loss. Clinical preliminary diagnoses were endometrial carcinoma or hyperplasia and ultrasound was supposed to be endometrial malignancy, hyperplasia or pyometra by radiologist. Histopathological examination of uterus revealed xanthogranulomatous endometritis. Since xanthogranulomatous endometritis may mimic endometrial malignancy clinically and pathologically as a result of the replacement of the endometrium and occasionally invasion of the myometrium by friable yellowish tissue composed of histiocytes, knowledge of this unusual inflammatory disease is needed for both clinicians and pathologists.

## 1. INTRODUCTION

Xanthogranulomatous endometritis (XGE) is an unusual inflammatory condition that peculiarly involves uterus [1–7]. XGE is similar both in macroscopic and microscopic appearance to xanthogranulomatous change occurring in other organs such as kidney and gall bladder, which are subject to the effects of chronic obstruction with subsequent infection [1, 2, 4, 7]. As XGEmay mimic endometrial malignancy clinically and pathologically as a consequence of the replacement of the endometrium and sometimes invasion of the myometrium by friable yellowish tissue composed of histiocytes, knowledge of this uncommon inflammatory disease is necessary for both clinicians and pathologists.

## 2. CASE REPORT

A 67-year-old female, mother of 5 children, complained two months history of post menopausal bleeding and weight loss without evidence of pain or fever. Physical examination findings were normal. Gynecologic examination revealed uterine prolapsus. Laboratory tests showed marked elevation of white blood cells (11800) and low hemoglobin value (9mg/dl). On transvaginal ultrasound, endometrium was measured 2.6 cm thick and marked heterogenic appearance with cystic hypoechoic areas in endometrium was detected. Those findings were supposed to be endometrial malignancy, hyperplasia or pyometra by radiologist. Endometrial culture and biopsy were performed but during biopsy very limited small fragmented tissue was obtained. Histological examination of this small fragmented tissue revealed abundant foamy histiocytes with variable amount of multinucleated giant cells, hemosiderin, and mixed inflammatory reaction. No evidence of normal endometrial glands, endometrial hyperplasia or carcinoma was found. Endometrial culture revealed no specific microorganism.

Total abdominal hysterectomy and bilateral salpingooopherectomy (TAH and BSO) was planned. During operation, intraoperative pathology consultation (frozen section) was performed. Onmacroscopic examination, TAH and BSO specimen was measured 10 × 8 × 5 cm. Endometrium was observed linear in some areas and irregular in other areas. Endometrium was measured 2 mm thick. Myometrial thickness was 12 mm. Both ovaries and uterine tubes were unremarkable. Whole endometrium was sampled for routine histopathological examination.

**Figure 1:**
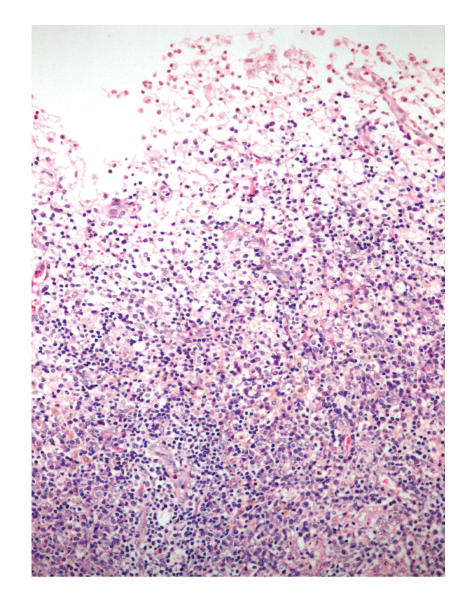
Histopathological features of endometrial samples include abundant foamy histiocytes and inflammatory cells (H and E).

**Figure 2:**
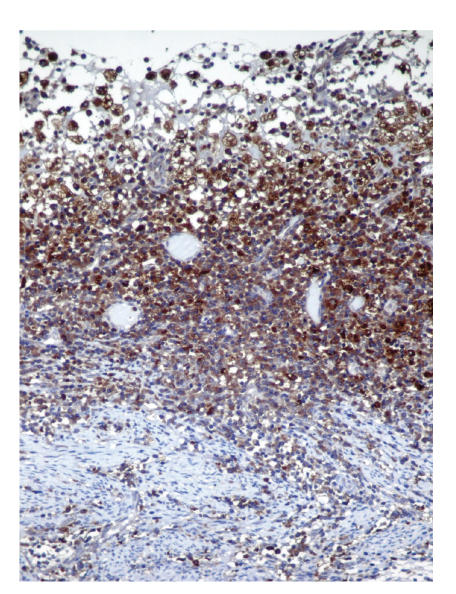
CD 68 positive histiocytes within the endometrium.

**Figure 3:**
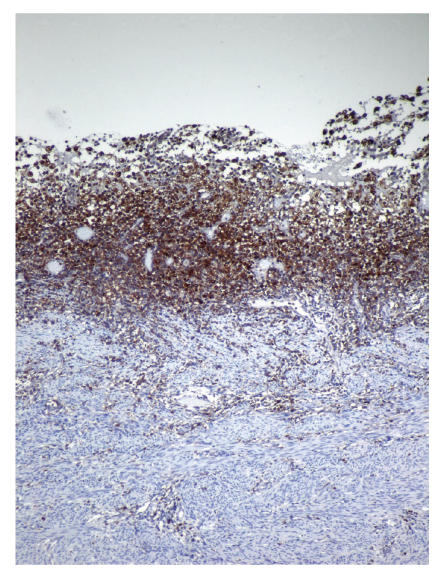
Mac-387 positive histiocytes within the endometrium.

**Figure 4:**
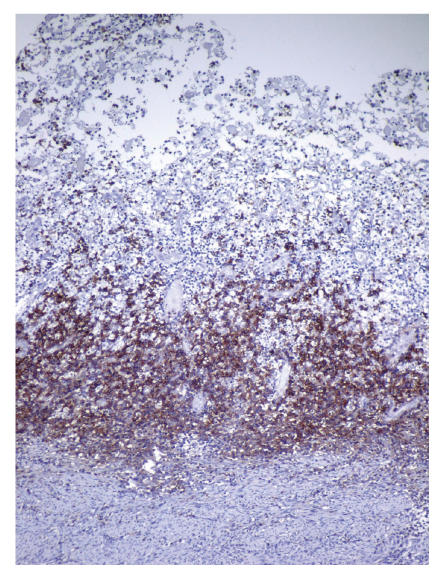
CD 138 positive plasma cells within the endometrial inflammation.

On histopathological examination of endometrial samples, abundant foamy histiocytes, mixed inflammatory reaction composed of polymorphonuclear leucocytes, plasma cells and lymphocytes, hemosiderin, calcification, and fibrosis were detected (see Figure 1). Neither of the endometrial samples showed hyperplasia or carcinoma. Additionally, chronic cervicitis and focal mucinous metaplasia of tubal epithelium with minimal chronic inflammation were detected. Special histochemical stains such as periodic acid schiff (PAS), Grocots methenamine silver (GMS), Gram stain, Prussian blue, and Von Cossa were applied on the endometrial samples. PAS, GMS, and Gram stain showed no specific microorganism; neither bacteria nor fungi. Prussian blue revealed intracytoplasmic hemosiderin accumulation in the foamy histiocytes. Von Cossa stain showed no calcium deposition within the endometrial inflammation. Immunohistochemically CD68 (1:40, mousemonoclonal, Neomarkers, Westinghouse, USA), Mac 387 (1:10, clone Mac 387, mouse monoclonal, DAKO, Denmark), CD 20 (for detecting B lymphocytes, 1:100, Clone L26, mouse monoclonal, Neomarkers, Westinghouse, USA), UCHL-1 (for detecting T lymphocytes, 1:100, mousemonoclonal, Neomarkers, Westinghouse, USA), and CD 138 (for detecting plasma cells, ready to use, mouse monoclonal, Neomarkers, Westinghouse, USA) were studied. The foamy cells were stained strongly positive for Mac 387 and CD 68 (see Figures 2 and 3). T and B lymphocytes and plasma cells were found within the endometrial inflammation with CD 20, UCHL-1, and CD 138, respectively Figure 4.

## 3. DISCUSSION

Although xanthogranulomatous inflammation is a distinguished histopathological entity affecting various organs, chiefly the kidney and gallbladder, XGE is very uncommon [1–4]. Up to now, only a small number of cases of xanthogranulomatous inflammation occurring in female genital tract have been described, most frequently affecting the endometrium and sometimes associated with endometrial carcinoma [1–3]. In 1978, Barua et al. described the first case of XGE without evidence of endometrial carcinoma or hyperplasia [4]. Two years later, Buckley and Fox described two cases and labeled them as “histiocytic endometritis” [7]. In 1983, Ashkenazy et al. described four cases with endometrial foamy cells. Two of their cases were presented with endometrial hyperplasia or carcinoma and the other two isolated XGE cases revealed hemosiderin in foamy cells [3]. In 1985, Pounder and Iyer reported a case of XGE associated with endometrial adenocarcinoma [1]. The foamy cells of their case contained brown pigment that supposed to be lipofuscin or hemosiderin [1]. In 1989, Blanco et al. reported a case of XGE and the foamy cells were described to be macrophages, components of a nonspecific inflammatory reaction, which have phagocytosed breakdown elements of retained endometrial hemorrhage [6]. In 1990, Russack and Lammers reported six XGE cases associated with endometrial carcinoma [2]. The pathogenesis of their cases was supposed to be associated with radiation therapy for endometrial carcinoma [2]. Recently, Noack et al. reported a case of XGE with lethal outcome [5]. They showed enterococci and *P. magnus* from the cultured uterine material. Barua et al. postulated infection with *E. coli* or *P. vulgaris* as a cause of XGE [4]. However, Buckley and Fox demonstrated no bacteria in their two cases [7]. The presented case showed no microorganism with special histochemical stains and culture.

Xanthogranulomatous cholecystitis and pyelonephritis develop on basis of inflammation, tissue necrosis and obstruction, and stenosis. The development of XGE may be influenced with various factors, including tumor bulk or death tumor cells due to radiation therapy or necrosis, presence of abundant amount of intrauterine hemorrhage and cervical stenosis or pre-existing vascular compromise such as atherosclerosis [1, 2]. Although these foamy cells that were infiltrating the endometrium were supposed to be histiocytes, their positive staining with histiocytic markers such as CD 68 and Mac 387 had not been proved before. Both T and B lymphocytes labeling with CD 20 and UCHL-1 were found within this lesion confirming poly clonality and definitely excluding lymphoma.

The coexistence of endometrial adenocarcinoma with XGE is very important for a diagnostic pathologist. The irregular and necrotic appearance of XGE may mimic carcinoma grossly. Histologically, the foamy histiocytes infiltrating themyometrium might bemisdiagnosed as clear cell carcinoma or sarcoma, although the cytological and immunohistochemical detail should easily resolve the issue. Awareness of XGE is important for pathologists and gynecologists since it may mimic malignancies [5]. However, the presence of XGE does not exclude the likelihood of an accompanying carcinoma, because of this possibility; we recommend the sampling of entire endometrium in XGE cases.
